# Perpendicular Magnetic Anisotropy in Heusler Alloy Films and Their Magnetoresistive Junctions

**DOI:** 10.3390/ma11010105

**Published:** 2018-01-11

**Authors:** Atsufumi Hirohata, William Frost, Marjan Samiepour, Jun-young Kim

**Affiliations:** 1 Department of Electronic Engineering, University of York, York YO10 5DD, UK; wf532@york.ac.uk (W.F.); marjan.samiepour@york.ac.uk (M.S.); 2 Department of Physics, University of York, York YO10 5DD, UK; junyoung.kim@york.ac.uk

**Keywords:** Heusler alloys, half-metallic ferromagnets, giant magnetoresistance, perpendicular magnetic anisotropy

## Abstract

For the sustainable development of spintronic devices, a half-metallic ferromagnetic film needs to be developed as a spin source with exhibiting 100% spin polarisation at its Fermi level at room temperature. One of the most promising candidates for such a film is a Heusler-alloy film, which has already been proven to achieve the half-metallicity in the bulk region of the film. The Heusler alloys have predominantly cubic crystalline structures with small magnetocrystalline anisotropy. In order to use these alloys in perpendicularly magnetised devices, which are advantageous over in-plane devices due to their scalability, lattice distortion is required by introducing atomic substitution and interfacial lattice mismatch. In this review, recent development in perpendicularly-magnetised Heusler-alloy films is overviewed and their magnetoresistive junctions are discussed. Especially, focus is given to binary Heusler alloys by replacing the second element in the ternary Heusler alloys with the third one, e.g., MnGa and MnGe, and to interfacially-induced anisotropy by attaching oxides and metals with different lattice constants to the Heusler alloys. These alloys can improve the performance of spintronic devices with higher recording capacity.

## 1. Introduction

Since the discovery of giant magnetoresistance (GMR) by Fert [1] and Grünberg [2] independently, magnetoresistive (MR) junctions have been used widely in many spintronic devices [3,4], e.g., a read head in a hard disk drive (HDDs) [5], and a cell in a magnetic random access memory (MRAM) [6]. The maximum GMR ratio achieved in a [Co (0.8)/Cu (0.83)]_60_ (thickness in nm) junction was reported to be 65% at 300 K [7]. Here, the MR ratio is determined by

MR ratio = Δ*R*/*R* = (*R*_AP_ − *R*_P_)/*R*_P_, (1)

where *R*_P_ and *R*_AP_ represent the resistance measured for parallel and antiparallel configurations of the ferromagnet magnetisations, respectively. In parallel, tunnelling magnetoresistance (TMR) [8] has been observed by utilising an oxide barrier instead of a non-magnetic spacer at room temperature (RT) [9,10], and have been improved its ratio very rapidly to 81% in a Co_0.4_Fe_0.4_B_0.2_ (3)/Al (0.6)-Ox/Co_0.4_Fe_0.4_B_0.2_ (2.5) (thickness in nm) junction at RT [11]. By replacing amorphous AlO_x_ with epitaxial MgO [12,13] as theoretically predicated [14,15], 604% TMR ratio has been achieved in a Co_0.2_Fe_0.6_B_0.2_ (6)/MgO (2.1)/Co_0.2_Fe_0.6_B_0.2_ (4) (thickness in nm) junction at RT [16]. Such drastic increase in the TMR ratio has increased the areal density of HDD by almost four times over the last decade, for example [3].

**Figure 1:**
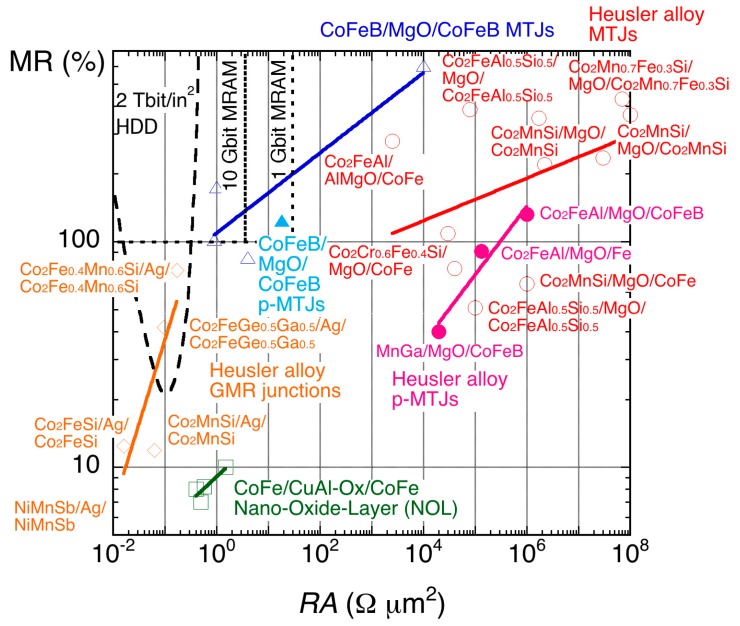
Relationship between magnetoresistance (MR) and resistance-area product (*RA*) of magnetic tunnel junctions (MTJs) with CoFeB/MgO/CoFeB (blue triangles), nano-oxide layers (NOL, green squares) and Heusler alloys (red circles) with in-plane (open symbols) and perpendicular magnetic anisotropy (closed symbols) together with that of giant magnetoresistive (GMR) junctions with Heusler alloys (orange rhombus). The target requirements for 2 Tbit/in^2^ hard disk drive (HDD) read heads as well as 1 and 10 Gbit magnetic random access memory (MRAM) applications are shown as purple and yellow shaded regions, respectively.

For further improvement in HDD and MRAM, it is critical to satisfy two criteria: (i) low resistance-area product (*RA*) and (ii) perpendicular magnetic anisotropy. The low *RA* is important to reduce power consumption and resulting unfavourable side effects, such as Joule heating and possible damage on spintronic devices. The perpendicular anisotropy is essential to achieve faster magnetisation switching [17,18] and to minimise stray fields from a MR junction and the associated cross-talk between the junction cells for MRAM. The recent development in MR ratios and *RA* is summarised in Figure 1. Figure 1 also includes the target requirements to achieve 1 Gbit MRAM, 10 Gbit MRAM and 2 Tbit/in^2^ HDD [19].

For the 1 Gbit MRAM, the junction cell diameter (fabrication rule) should be <65 nm with *RA* < 30 Ω·µm^2^ and MR ratio > 100% [19]. For the 10 Gbit MRAM, the cell diameter should be <20 nm with *RA* < 3.5 Ω·µm^2^ and MR ratio >100%. Here, low *RA* is required to satisfy the impedance matching [20] with a transistor attached to one MRAM cell and a large MR ratio is essential to maintain a signal-to-noise ratio allowing for a read-out signal voltage to be detected by a small-current application. In order to achieve these requirements, intensive research has been performed on the CoFeB/MgO/CoFeB junctions. As shown as open triangles with a blue fit in Figure 1, in-plane CoFeB/MgO/CoFeB magnetic tunnel junctions (MTJs) have successfully satisfied the requirement for the 10 Gbit MRAM by achieving *RA* = 0.9 Ω·µm^2^ and TMR = 102% at RT [21]. Later, a perpendicularly-magnetised MTJ (p-MTJ) also achieved the requirement for the 1 Gbit MRAM with *RA* = 18 Ω·µm^2^ and TMR = 124% at RT [22], which requires further improvement for the 10 Gbit MRAM target. Such MTJs will replace the current-generation 256 Mbit MRAM with perpendicular magnetic anisotropy produced by Everspin [23].

For the 2 Tbit/in^2^ HDD, on the other hand, the MTJs cannot be used as the requirement for *RA* is almost one order of magnitude smaller than that for the 10 Gbit MRAM [24]. One attempt is nano-oxide layers (NOL), which restrict the current paths perpendicular to the GMR stack by oxidising a part of the Cu or Al spacer layer [25]. In a Co_0.5_Fe_0.5_ (2.5)/Al-NOL/Co_0.5_Fe_0.5_ (2.5) junction, *RA* = 0.5~1.5 Ω·µm^2^ and MR = 7~10% at RT has been achieved. These values are below the requirement for the 2 Tbit/in^2^ HDD, and hence further improvement in GMR or TMR junctions are crucial.

## 2. Heusler-Alloy Junctions

For the further improvement in the MR junctions to meet the requirements for 10 Gbit MRAM and 2 Tbit/in^2^ HDD, a half-metallic ferromagnet needs to be developed to achieve 100% spin polarisation at the Fermi energy at RT, leading to an infinite MR ratio using Equation (1). The half-metallicity is induced by the formation of a bandgap only in one of the electron-spin bands. There have been five types of half-metallic ferromagnets theoretically proposed and experimentally demonstrated to date: (i) oxide compounds (e.g., rutile CrO_2_ [26] and spinel Fe_3_O_4_ [27]); (ii) perovskites (e.g., (La,Sr)MnO_3_ [28]); and, (iii) magnetic semiconductors, including Zinc-blende compounds (e.g., EuO and EuS [29], (Ga,Mn)As [30] and CrAs [31]) and (iv) Heusler alloys (e.g., NiMnSb [32]). Magnetic semiconductors have been reported to show 100% spin polarisation due to their Zeeman splitting in two spin bands. However, their Curie temperature is still below RT [33]. Low-temperature Andreev reflection measurements have confirmed that both rutile CrO_2_ and perovskite La_0.7_Sr_0.3_MnO_3_ compounds possess almost 100% spin polarisation [34], however, no experimental report has been proved the half-metallicity at RT. As the most promising candidate for the RT half-metallicity, a Heusler alloy has been studied extensively as detailed in the following sections [35,36,37].

### 2.1. Heusler Alloys

#### 2.1.1. Crystalline Structures

**Figure 2:**
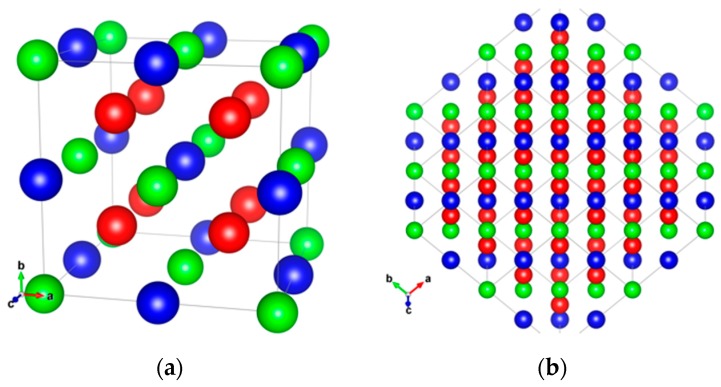
(**a**) Schematic unit cell of the *L*2_1_-ordered full-Heusler alloy consisting of X_2_YZ atoms (X: red, Y: blue and Z: green); (**b**) (110) plane projection of the corresponding Heusler alloy.

Since the initial discovery of ferromagnetism in a ternary Cu_2_MnAl alloy, consisting of non-magnetic elements by Heusler in 1903 [38], the Heusler alloys have been studied for various applications, including magnetic refrigeration [39] and shape memory [40]. The Heulser alloys are categorised into two types: full- and half-Heusler alloys in the forms of X_2_YZ and XYZ, respectively, where X and Y are transition metals and Z is a semiconductor or non-magnet. Figure 2a shows a schematic crystalline structure of the full-Heusler alloy in the perfectly ordered *L*2_1_-phase. By mixing Y and Z, the alloy forms the partially-mixed *B*2-phase, while further mixing among X, Y, and Z makes the fully-disordered *A*2-phase. By replacing a half of X atoms with Y-site atoms, Y atoms with Z-site atoms and Z atoms with X-site atoms, inverse Heusler alloys in the *D*0_3_-phase can be formed. The removal of a half of the X atoms makes the half-Heusler alloys in the *C*1*_b_*-phase. Additionally, a part of the constituent atoms can be replaced with the other atoms, allowing for controlling their crystalline and magnetic properties, such as lattice constants, magnetic moments, and magnetic anisotropy.

Due to the above complicated crystalline structures for the Heusler alloys, they require very high temperature (typically >1000 K in the bulk form and >650 K in the thin-film form) for their crystallisation [41]. This prevents the Heusler alloys to be used in spintronic devices. Recently, layer-by-layer growth in the Heusler alloy (110) plane (see Figure 2b) has been reported to decrease the crystallisation energy, i.e., the annealing temperature, by over 50% [42]. A similar crystallisation process has been demonstrated at higher temperature to uniformly crystallise the Heusler-alloy films [43].

#### 2.1.2. Magnetic Properties

The robustness of the half-metallicity depends on the size and definition of the bandgap formed in one electron-spin band in the vicinity of Fermi energy. The bandgap is formed by the strong *d*-band hybridisation between the two transition metals of X and Y, according to ab initio calculations [34]. Typically, the bandgap of 0.4~0.8 eV is expected to be formed at 0 K [36]. At a finite temperature, however, the bandgap becomes smaller and the edge definition of the gap becomes poorly-defined. The bandgap has been measured by detecting photon absorption of circularly-polarised infrared light with energy corresponding to the bandgap [44].

**Figure 3:**
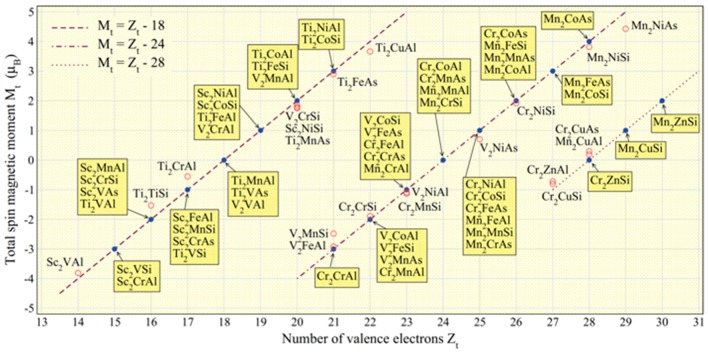
Total spin magnetic moments per unit cell (*M*_t_/f.u.) as a function of the total number of valence electrons in the unit cell for major Heusler alloys. The lines represent three different forms of the generalised Slater-Pauling curves [45].

The other advantage of the Heusler alloys is their controllability of their magnetic properties, such as their saturation magnetisation and Curie temperature. The total spin moments per Heusler alloy formula unit (f.u.) (*M*_t_) have been reported to follow the generalised Slater-Pauling curve as *M*_t_ = *Z*_t_ − 24 (full-Heusler) and *M*_t_ = *Z*_t_ − 18 (half-Heusler), where *Z*_t_ is the total number of valence-band electrons (see Figure 3) [45]. The atomic substitutions of any constituent atoms in the Heusler alloys can continuously change their magnetic moments and allows for customising the alloys for a specific application. There are over 2500 combinations to form Heusler alloys [36], among which a few tens of alloys have been reported to become half-metallic ferromagnets according to theoretical calculations. The atomic substitution further increase the applicability of the alloys for custom design.

### 2.2. Heusler Alloy Junctions with In-Plane Magnetic Anisotropy

#### 2.2.1. Tunnelling Magnetoresistive Junctions

(1) Co_2_(Cr,Fe)Z

A pioneering work on a Heusler-alloy junction has been carried out by Block et al. [46]. They have reported a large negative MR ratio at RT in a quarternary full-Heusler Co_2_Cr_0.6_Fe_0.4_Al alloy, which experimentally demonstrates the controllability of the magnetic properties of the alloys by substituting their constituent elements. They report 30% MR at RT with pressed powder compacts, which acts as a series of MTJs. The Co_2_(Cr,Fe)Al alloys have then been used in MTJs in their polycrystalline form. A MTJ with the structure of Co_2_Cr_0.6_Fe_0.4_Al/AlOx/CoFe shows 16% TMR at RT [47], which is later improved up to 19% at RT by the barrier optimisation [48].

Recently, an epitaxial *L*2_1_-Co_2_Cr_0.6_Fe_0.4_Al film sputtered onto MgO(001) substrate has been adopted for a fully epitaxial MTJ, consisting of Co_2_Cr_0.6_Fe_0.4_Al/MgO/CoFe, showing 42% at RT (74% at 55 K) [49]. Even though this film possesses the crystalline relationship Co2Cr0.6Fe0.4Al(001)[100]||MgO(001)[110], the magnetic moment is estimated to be 3.3 µ_B_/f.u., which is smaller than the calculation (3.7 µ_B_/f.u.) [50]. This indicates that the film contains an atomically disordered phase, which is also suggested from the decrease in the TMR ratios that are measured below 55 K. Further optimisation results in the TMR ratio to become 109% at RT and 317% at 4 K with *RA* ~ 3 × 10^4^ Ω·µm^2^ [51].

The half-metallicity of the Co_2_Cr_1-*x*_Fe*_x_*Al full-Heusler alloys has been found to be robust against the atomic disorder using first-principles calculations by Shirai et al. [52]. In the Co_2_CrAl alloys, the atomic disorder between Cr and Al, which eventually deforms the crystalline structure from *L*2_1_ into *B*2 at a disorder level of 0.5, maintains the very high spin polarisation (*P*) of 97% for *L*2_1_ and 93% for *B*2. The Co-Cr type disorder, however, destroys the half-metallicity rapidly, i.e., *P* to zero at a disorder level of 0.4 and *M*_t_ to be 2.0 µ_B_/f.u. at the full disorder. For the Fe substitution *x* with Cr, high *P* is calculated to be maintained above 90% up to *x* = 0.35. Similarly, the CrFe-Al type disorder preserves both spin polarisation and the magnetic moment to be above 80% and 3.7 µ_B_/f.u., respectively, up to the disorder level of 0.5, while the Co-CrFe disorder eliminates *P* at the disorder level of 0.3. These findings may explain the decrease in the measured TMR ratios as compared with the theoretically predicted value due to the interfacial disorder.

Strain also affects the half-metallicity in the Co_2_CrAl alloy, according to calculations [53]. *P* stays 100% in the lattice strain range between 1 and +3%, and is even higher than 90% up to +10% strain. The bandgap is also maintained against the strain and can be maximised under +3% strain. *P* also remains 100% against the tetragonal distortion in the range of ±2%, which is a great advantage for the epitaxial growth study on a GaAs substrate [54] and the other seed layers.

Unlike Co_2_CrAl, Co_2_FeAl is not theoretically predicted to be half-metallic [50]. Even so, Epitaxial Co_2_FeAl films are grown on GaAs(001) with the relationship Co2FeAl(001)[110]||GaAs(001)[110]. Accordingly, an epitaxial full Heusler Co_2_FeAl film with the *L*2_1_ structure is also applied for a MTJ but shows only 9% TMR at RT [54]. These small TMR ratios may be caused by the selective oxidation at the interface between the Heusler films and the oxide barriers. The TMR ratios have been increased to 330% at RT (700% at 10 K) with *RA* = 1 × 10^3^ Ω·µm^2^ in a MTJ with Co_2_FeAl/MgO/Co_0.75_Fe_0.25_ by utilising the Δ_1_-band connection between Co_2_FeAl and MgO [55]. Using a MgAlOx barrier instead of MgO to maintain the Δ_1_-band connection and to make better lattice matching with *B*2-Co_2_FeAl, TMR ratios are found to be increased to 342% at RT (616% at 4 K) with *RA* = 2.5 × 10^3^ Ω·µm^2^ [56]. The departure of the TMR ratios from theoretically predicted almost infinity may also be due to the interfacial atomic disorder, due to the presence of a light element of aluminium.

By replacing a half of Al with Si in Co_2_FeAl to stabilise the crystallisation, MTJs with an oriented MgO barrier for which TMR ratios of 175% have been achieved at RT when using *B*2-Co_2_FeAl_0.5_Si_0.5_ [57]. Using *L*2_1_-Co_2_FeAl_0.5_Si_0.5_, the TMR ratios of 386% at RT and 832% at 9 K with *RA* = 80 × 10^3^ Ω·µm^2^ has been reported later [58]. The decrease in the TMR ratio with increasing temperature is much faster than the temperature dependence of the magnetisation *T*^3/2^, suggesting that a small fraction of atomically disordered phases cannot be ignored in the spin-polarised electron transport at finite temperatures [59]. The elimination of such disordered interfacial phases improves the TMR ratios further and realises the half-metallicity at RT.

Theoretical calculations suggest that the interface states within the half-metallic bandgap formed at the half-metal/insulator interfaces prevent the highly spin-polarised electron transport [60]. This is because the tunneling rate is slower than the spin-flip rate, and therefore the interface states for the minority spins are effectively coupled to the metallic spin reservoir of the majority spin states. In order to avoid the spin-flip scattering, a sharp interface without the interface states is crucially required.

(2) Co_2_MnZ

Another pioneering work on the growth of full Heusler alloy films has been performed for a Co_2_MnGe/GaAs(001) hybrid structure by Ambrose et al. [61]. They achieve an epitaxial Co_2_MnGe film with a slightly enhanced lattice constant as compared with bulk. *M*_t_ is estimated to be 5.1 µ_B_/f.u., which almost perfectly agrees with the bulk and theoretically predicted value from the generalised Slater-Pauling curve. Consequently, systematic study has been widely carried out over Co_2_Mn-based full Heusler alloys to realise the RT half-metallicity: Co_2_MnAl [62,63], Co_2_MnSi [64,65], Co_2_MnGa [66], and Co_2_MnSn [64]. For example, an epitaxial Co_2_MnAl film has been grown on a Cr buffer layer by sputtering with the crystalline relationship Co_2_MnAl(001)[110]||Cr(001)[110]||MgO(001)[100] with the *B*2 structure [60]. For Co_2_MnSi, the *L*2_1_ structure has been deposited by using both dc magnetron sputtering [67] and MBE [68].

Calculations imply that the strain induced can control the half-metallicity in the Co_2_MnZ alloys. For Co_2_MnSi, for example, the lattice compression of 4% increases the bandgap by 23%, and a similar behavior is expected for the other alloy compounds [69]. Similarly, ±2% change in the lattice constant preserves the half-metallicity in the Co_2_MnZ alloys [33].

A MTJ with an epitaxial *L*2_1_-Co_2_MnSi film has been reported to show very large TMR ratios of 70% at RT and 159% at 2 K with *RA* = 10^6^ Ω·µm^2^ [70]. These values are the largest TMR ratios obtained in a MTJ employing a Heusler-alloy film and AlO_x_ barrier. This is purely induced by the intrinsic *P* of the Heusler electrodes. Similarly, a MTJ with Co_2_MnAl/AlO_x_/CoFe shows 40% TMR at RT [63], followed by the further improvement up to 61% at RT (83% at 2 K) [71]. All of these Heusler films in the MTJs have been reported to be *B*2 structure. By comparing the TMR ratios at RT with those at low temperature, the TMR ratios are found to show very weak temperature dependence as similarly observed for a conventional metallic MTJ. On the contrary, a MTJ with a highly ordered Co_2_MnSi film shows strong temperature dependence; 33% at RT and 86% at 10 K [72], and 70% at RT and 159% at 2 K [70]. Such rapid decrease in the TMR ratio with an increasing temperature is similar to that observed in MTJs with Co_2_(Cr,Fe)Al.

By replacing AlO_x_ with MgO, a fully epitaxial MTJ, consisting of Co_2_MnSi/MgO/Co_2_MnSi, has been reported to achieve much higher TMR ratios, 217% at RT (753% at 2 K) [73] and 236% at RT (1135% at 4 K), but with larger *RA* of 3 × 10^7^ Ω·µm^2^ [74]. Further improvements in the TMR ratio to be 354% at RT (1995% at 4 K) have been achieved in the same system [75], followed by 366% at RT (2110% at 4 K) with *RA* = 10^8^ Ω·µm^2^ [76]. Partial substitution of Mn with Fe in these MTJs to form Co_2_Mn_0.73_Fe_0.27_Si, TMR ratios are increased to 429% at RT (2610% at 4 K) with *RA* = 7 × 10^7^ Ω·µm^2^ [77], which is the largest TMR ratio reported to date. A similar MTJ with Co_2_MnGe/MgO/Co_2_MnGe has been fabricated to show similar TMR ratios of 220% (650% at 4 K), but with large *RA* of 2.2 × 10^6^ Ω·µm^2^ [78].

(3) Ni_2_MnZ

Even though Ni_2_MnZ alloys are not predicted to become half-metallic ferromagnets by calculations, detailed studies on epitaxial growth on GaAs and InAs has been reported by Palmstrøm et al. [79]. By using a Sc_0.3_Er_0.7_As buffer layer on GaAs(001), both Ni_2_MnAl [80] and Ni_2_MnGa [81,82] films are epitaxially grown with the crystalline relation-ship Ni2MnGa(001)[100]||GaAs(001)[100] [83]. All the films are slightly tetragonally elongated along the plane normal as compared with the bulk values due to the minor lattice mismatch with the semiconductor substrates. First-principles calculations demonstrate that a broad energy minimum of tetragonal Ni_2_MnGa can explain stable pseudomorphic growth of Ni_2_MnGa on GaAs despite a nominal 3% lattice mismatch [84].

(4) Half-Heusler

After the first theoretical prediction of the half-metallicity of the half-Heusler NiMnSb alloy [30], this alloy has been intensively investigated to confirm its half-metallicity experimentally. *M*_t_ and the bandgap are calculated to be approximately 3.99 µ_B_/f.u. and 0.5 eV [85], respectively, resulting in calculated spin polarisation of 99.3% [86]. Epitaxial NiMnSb(001) growth on GaAs(001) has also been studied systematically by van Roy et al. [87]. An epitaxial half Heusler NiMnSb film has been first used as an electrode in a MTJ, showing 9% TMR at RT [88].

#### 2.2.2. Giant Magnetoresistive Junctions

Similar to the TMR junctions as discussed in Section 2.1.1, GMR junctions with Heusler-alloy films have been studied over the last decades. For example, a GMR junction, consisting of Co_2_MnGe (6)/V (1.6)/Co_2_MnGe (3)/Fe (0.3)/ZnSe (50)/GaAs(001) (thickness in nm) have been fabricated and measured along the two [110] directions [89]. The GMR ratio is measured to be less than 1%. Since then, a series of GMR juncstions have been designed and evaluated. An epitaxial film is deposited on a MgO(001) substrate with the crystalline relationship Co_2_Cr_0.6_Fe_0.4_Al(001)[100]||MgO(001)[110]. Here, by repeating [Co_2_Cr_0.6_Fe_0.4_Al (10)/Cu (2.5)/Fe_0.1_Co_0.9_ (8.1)] stack, current-in-the-plane (CIP) GMR has been measured, showing only 2% GMR at RT (4% at 15 K) [90]. Further enhancement has been reported in CPP-GMR devices, consisting of Co_2_FeAl_0.5_Si_0.5_ (2.5)/Ag (5)/Co_2_FeAl_0.5_Si_0.5_ (2.5) (thickness in nm), to be GMR ratios and *RA* of 34% and 8 × 10^−3^ Ω·μm^2^ at 290 K (80% at 14 K) [91].

Simultaneously, a large GMR ratio of 42% has been reported using Co_2_FeGe_0.5_Ga_0.5_/Ag/Co_2_FeGe_0.5_Ga_0.5_ junctions [92]. Theoretically, a larger GMR ratios are expected, e.g., 90% and ~60% for *L*2_1_- and *B*2-Co_2_MnAl/Ag/Co_2_MnAl junctions, respectively [93]. These junctions clearly have the capability of being used as a next-generation read head.

Similar argument can be applied for the GMR junctions with the half-Heusler-alloy films. PtMnSb films are deposited on Al_2_O_3_(0001) by sputtering to form spin-valve structures, PtMnSb(111)/CuMnSb(111)/PtMnSb(111)/MnFe, showing 0.47% GMR at RT [94]. This may also be due to the empty site disorder. Calculations suggest the decrease in the surface spin polarisation dependent upon the terminated layers: spin polarisation of ~46% and 22% for the MnSb and Pt termination, respectively [95]. The other half Heusler alloy CoMnSb shows a similar decrease in the surface spin polarisation and the bandgap change by the strain: +2% and −2% lattice deformation shifts the bandgap by 0.8 eV and +0.9 eV, respectively [96]. Recently, current-perpendicular-to-the-plane (CPP)-GMR ratios of 8% at RT (21% at 4 K) has been reported in fully-epitaxial NiMnSb (20)/Ag (5)/NiMnSb (7) (thickness in nm) junctions with the (001) orientation [97]. The junctions achieve *RA* = (26 ± 1) × 10^−3^  Ω·μm^2^, which is highly advantageous for device applications with further improvement in the GMR ratios. By repeating two sets of epitaxial GMR junctions, consisting of NiMnSb (9)/Ag (5)/NiMnSb (3)/Ag (5)/NiMnSb (9) (thickness in nm), an increase in the CPP-GMR ratio up to 11% (41% at 4 K) has been reported later [98]. Here, *RA* is found to be reduced to 3.9 × 10^−3^  Ω·μm^2^, which is favourable for device application.

### 2.3. Heusler Alloy Junctions with Perpendicular Magnetic Anisotropy

#### 2.3.1. Tunnelling Magnetoresistive Junctions

**Figure 4:**
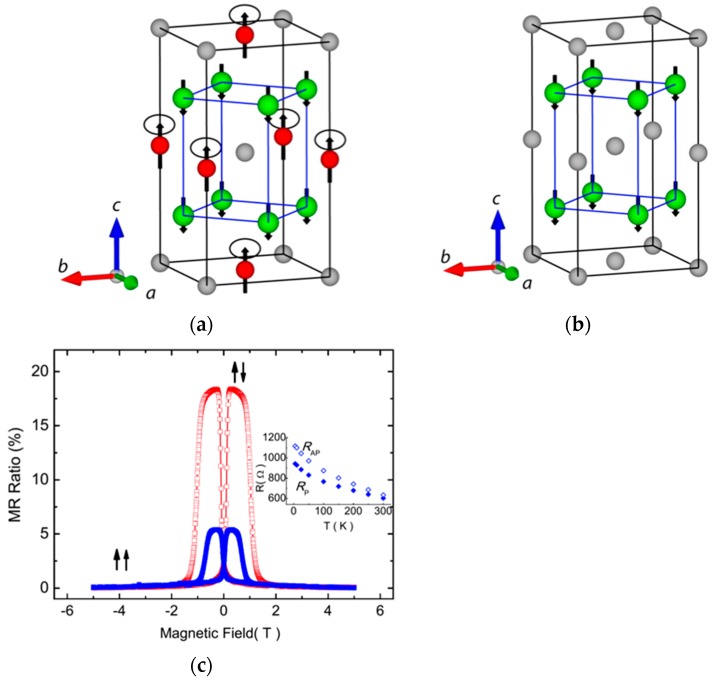
(**a**) The ferrimagnetic structure of *D*0_22_ Mn_3_Ga or *D*0_22_ Mn_3_Ge. There is overall *c*-axis anisotropy but the structure is noncollinear and the 2*b* sublattice has a soft, in-plane component, which is indicated by small circles; (**b**) Ferromagnetic structure of *L*1_0_ MnGa. The Mn atoms in the 4d positions all couple ferromagnetically [104]; (**c**) Perpendicular magnetoresistance (MR) loops for Mn_0.62_Ga_0.38_ (30)/Mg (0.4)/MgO (1.8)/CoFeB (1.2) (thickness in nm) measured at 300 K (blue solid squares) and 5 K (red open squares). The inset shows the temperature dependence of parallel and antiparallel resistances (*R*_P_ and *R*_AP_) [102].

By replacing Y atoms with X atoms, binary Heusler alloys can be formed. For example, Mn_3_Ga shows ferrimagnetic behaviour in the tetragonal *D*0_22_-phase with perpendicular magnetic anisotropy, as schematically shown in Figure 4a,b. The ferrimagnetic Mn_3_Ga has been reported to possess a large uniaxial anisotropy of 1 × 10^7^ erg/cm^3^ [99] and high Curie temperature of around 770 K [100]. Mn_3_Ga has been used in a MTJ, consisting of Mn_3_Ga/MgO/CoFe and has shown 9.8% TMR at 300 K with the perpendicular anisotropy of 1.2 × 10^7^ erg/cm^3^ [101]. The TMR ratio has then been improved by adjusting the Mn-Ga composition to be 40% at RT for the MTJ, consisting of Mn_0.62_Ga_0.38_ (30)/Mg (0.4)/MgO (1.8)/CoFeB (1.2) (thickness in nm) (see Figure 4c) [102]. This improvement may be due to the increase in the perpendicular anisotropy to be 5 × 10^6^ erg/cm^3^ in a similar MTJ [103], which is almost the same with that for the film reported above. However, the MTJ has 20 × 10^3^ Ω·µm^2^, which requires further reduction for the spintronic device applications.

By inserting Co_2_MnSi between Mn-Ga and MgO, the perpendicular anisotropy of the Mn-Ga layer can induce perpendicular anisotropy in the half-metallic Co_2_MnSi layer, which is expected to achieve a large TMR ratio. Experimentally, TMR ratios of 10% at RT and 65% at 10 K have been achieved [105], which is smaller than the Mn-Ga/MgO/Mn-Ga junctions, as above. Additionally, the Co_2_MnSi magnetisation is in tilted states during the reversal process, which makes the TMR curves to be not well-defined.

**Figure 5:**
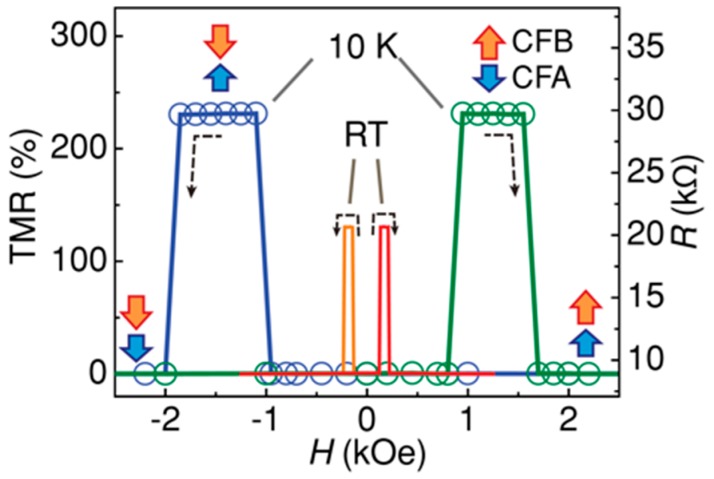
Tunneling resistance, *R*, as a function of out-of-plane magnetic field, *H*, measured at RT and 10 K for a p-MTJ, consisting of Co_2_FeAl (1.2)/MgO (1.8)/Fe (0.1)/CoFeB (1.3) (thickness in nm). Wide arrows illustrate the magnetisation states (P or AP) of bottom and top electrodes. The dashed lines with arrows represent sweeping directions of the magnetic fields with different traces. The directions of the magnetisations of the bottom and top electrodes were determined from *M*–*H* loops by checking the differences in magnetic moments and switching fields, respectively [107].

Similar to the CoFeB/MgO/CoFeB systems, as described in Section 1, perpendicular anisotropy has been induced by attaching a MgO tunnel barrier. In a p-MTJ, consisting of Co_2_FeAl/MgO/Co_0.2_Fe_0.6_B_0.2_, a TMR ratio of 53% has been reported at RT (see Figure 5) [106]. By inserting a 0.1-nm-thick Fe (Co_0.5_Fe_0.5_) layer between the MgO and Co_0.2_Fe_0.6_B_0.2_ layers, the TMR ratio was significantly enhanced to 91% (82%), due to the improved interface. The corresponding *RA* is 1.31 × 10^5^ Ω·µm^2^. By further improving the MTJ quality, consisting of Co_2_FeAl (1.2)/MgO (1.8)/Fe (0.1)/CoFeB (1.3) (thickness in nm), it has been reported to show TMR = 132% and *RA* = 1 × 10^6^ Ω·µm^2^ at RT [107].

A perpendicularly magnetised seed layer has also been used to induce perpendicular anisotropy onto the Heusler-alloy films. For example, a MTJ stack with *L*1_0_-CoPt/Co_2_MnSi/MgO/FePt has been demonstrated [108], as similarly reported in a conventional CoFeB/MgO/CoFeB junctions.

#### 2.3.2. Giant Magnetoresistive Junctions

**Figure 6:**
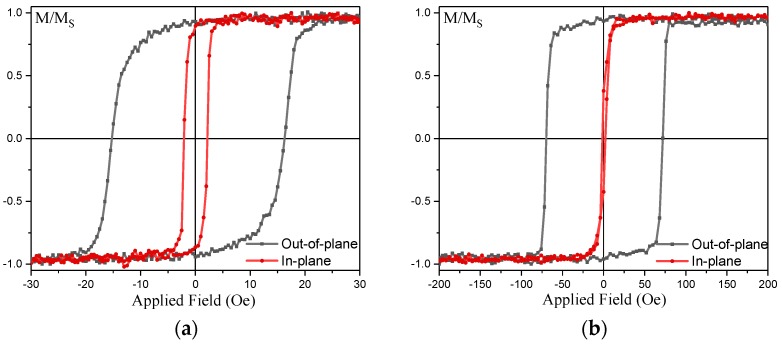
(**a**) Magnetisation curves of a Co_2_FeSi film grown on a V seed layer, consisting of Si sub./V (25)/Co_2_FeSi (4)/V (1.5)/Ru (3) (thickness in nm) measured under the magnetic field application in-plane (red) and perpendicular to the plane (blue); (**b**) Magnetisation curves of a Co_2_FeSi film grown on a W seed layer, consisting of Si sub./W (25)/Co_2_FeSi (4)/W (1.5)/Ru (3) (thickness in nm) measured under the magnetic field application in-plane (red) and perpendicular to the plane (black).

Recently, body-centred cubic (bcc) seed layers have been used to minimise the interfacial mixing with face-centred cubic (fcc) Heusler-alloy layer. For a bcc vanadium seed layer, X-ray analysis shows that 25-nm-thick vanadium introduces a strong (110) orientation in the Co_2_FeSi Heusler alloy [109]. The *B*2-texture of the Co_2_FeSi is found to match that of the vanadium proving that the texture is defined by the seed layer. Reduction of the Co_2_FeSi thickness is found to result in a reduction in the strength of the in-plane anisotropy, as expected from the cubic nature. Since the perpendicular magnetic anisotropy (PMA) is induced at the interface between the Co_2_FeSi and vanadium, a second vanadium interface is added and found to increase the observed PMA. Further reduction in the thickness of the Co_2_FeSi layer lead to an increase in the PMA where 4-nm-thick Co_2_FeSi exhibited a strong PMA (see Figure 6a). Here, the magnetic moment of the Co_2_FeSi layers all fell short of the bulk value with the saturation magnetisation (*M*_S_) of 700~800 emu/cm^3^. This may indicate magnetic dead layers at the interfaces due to roughness or intermixing; or could be due to a lack of full *L*2_1_-ordering resulting in a drop in net moment.

Vanadium and tungsten are similar materials in that both are transition metal elements, which crystallise in a bcc structure. They have similar lattice parameters of *a*_V_ = 0.3030 and *a*_W_ = 0.31648 nm, leading to 3.3% and 17% strain in Co_2_FeSi, respectively. Tungsten is, however, of much lower bulk resistivity with a value of 5.6 × 10^−6^ Ω·cm [110], which is around half the value for vanadium to be 1.9 × 10^−5^ Ω·cm [111]. As such, tungsten should give similar if not superior results to vanadium as a seed layer.

Accordingly, tungsten layers of 10~20 nm are deposited under 5-nm-thick Co_2_FeSi, resulting in the (110) texture in Co_2_FeSi, as similarly observed for the V seed samples. However, the W seed layer is found to be heavily oxidised [112]. X-ray reflectivity (XRR) indicates a smooth film with low interfacial roughness of 0.4 nm for W/WO_x_/Co_2_FeSi, which is comparable with 0.5 nm for V/Co_2_FeSi. The sample with a 20 nm W/WO_x_ seed layer exhibited clear in-plane anisotropy with a typical out-of-plane hard axis loop. The value of the anisotropy is low at only 1.58 × 10^4^ erg/cm^3^. This low value is due to the low value of *M*_S_ of ~400 emu/cm^3^. The 10 nm thick W/WO_x_ sample, however, exhibited a strong PMA in the Co_2_FeSi layer.

In an attempt to improve the quality of the tungsten seed layers, high temperature growth was utilised. The substrate is preheated to 673 K before deposition of 20 nm of tungsten. The resulting film shows a drastic reduction in oxidation with strongly crystallised tungsten. However, there is a lack of global texture as demonstrated by the multiple phases of tungsten. Scherrer analysis of the (110) peak gives an approximate crystallite size of 9 nm. The magnetisation of the W/Co_2_FeSi sample is measured to be 400 emu/cm^3^ with the perpendicular anisotropy of 8 × 10^5^ erg/cm^3^. These properties are summarised in Table 1.

## 3. Towards Device Implementation

**Figure 7:**
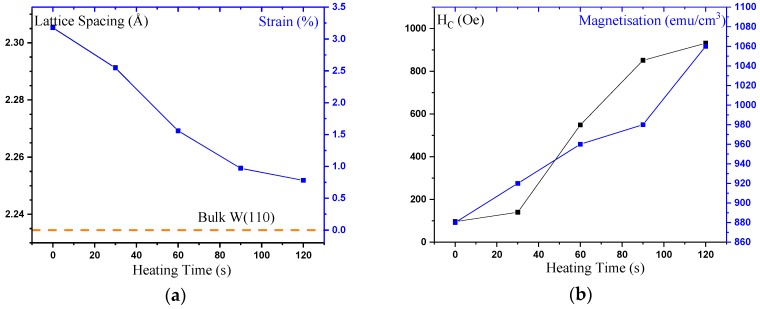
(**a**) W lattice constants calculated from the W(110) peak positions measured by X-ray diffraction for the W-seed sample, consisting of Si sub./W (10)/Co_2_Fe Al_0.5_Si_0.5_ (12.5)/W (1.2)/Co_2_Fe Al_0.5_Si_0.5_ (2.5)/Ta (2) (thickness in nm), as a function of pre-growth heating between 300 ≤ *T* ≤ 370 K. The bulk value is shown as a reference. (**b**) Corresponding evolution of saturation magnetisation and coercivity (*H*_c_) calculated from magnetisation curves.

Since the crystalline plane induced by the bcc seed layers is (110), which is a favourable orientation to promote the layer-by-layer crystallisation, low-temperature crystallisation has been demonstrated with PMA [115]. Samples consisting of W (10)/Co_2_FeAl_0.5_Si_0.5_ (12.5)/W (1.2)/Co_2_Fe Al_0.5_Si_0.5_ (2.5)/Ta (2) (thickness in nm) have been deposited with pre-growth heating at 300 ≤ *T* ≤ 370 K. Increasing temperature is found to cause a large increase in the crystallinity in the W(110) direction. As the heating time is increased, the position of the peak relaxed towards the bulk location, as shown in Figure 7a, corresponding a change in lattice spacing Δ*d* = (0.0053 ± 0.0001) nm out-of-plane, i.e., a change in strain of Δ*s* = (−2.4 ± 0.1) %. The position of the Heusler-alloy peak is not changed by increased deposition heating time. However, the intensity of the reflection increased significantly, indicating an increased crystallisation, as expected.

Magnetic characterisation of the samples is performed under both in- and out-of-the-plane fields. All of the samples with heated substrates showed perpendicular anisotropy. Figure 7b shows the coercivities (*H*_C_) and saturation magnetisations (*M*_S_) for the samples. *H*_C_ and *M*_S_ both increase monotonically with substrate temperature in agreement with the XRD data. The increased moment is due to the increase in the crystallisation of the material. After *T* = 305 K (30 s) the loop squareness decreases from *M*_R_/*M*_S_ = 1, but remains high (>0.8) up to *T* = 370 K (120 s). *M*_S_ reaches almost 1060 emu/cm^3^, which is almost 85% of the theoretically predicted value, and it is ideal for device implementation due to the low-temperature crystallisation.

**Figure 8:**
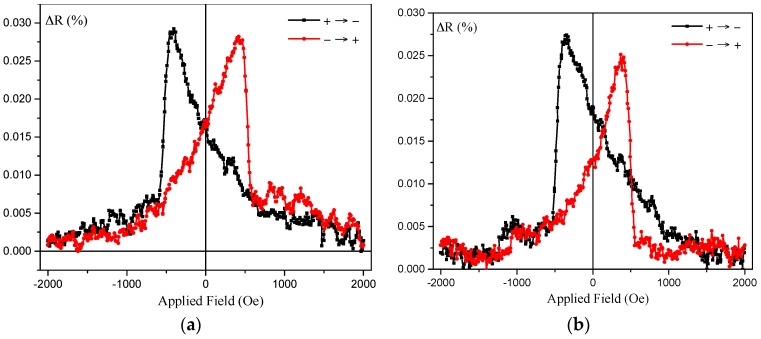
(**a**) Current-perpendicular-to-the-plane giant magnetoresistance (CPP-GMR) effect measured under perpendicular fields for the (**a**) V- and (**b**) W-seed Co_2_FeSi junctions, consisting of W (10)/Co_2_FeAl_0.5_Si_0.5_ (12.5)/Ag(3)/Co_2_FeAl_0.5_Si_0.5_ (5) (thickness in nm) with dimensions of (**a**) (1000 × 500) μm^2^ and (**b**) (150 × 100) μm^2^.

Due to band-structure matching silver makes an ideal conduction layer for Heusler alloy CPP-GMR devices. A 3 nm thick layer of Ag was deposited into a device structure Si sub./W (10)/Co_2_FeAl_0.5_Si_0.5_ (12.5)/Ag (3)/Co_2_FeAl_0.5_Si_0.5_ (5)/Ru (3) where thicknesses are in nm. These were patterned using e-beam lithography into elliptical devices with dimensions from (1000 × 500) nm^2^ to (150 × 100) nm^2^. The ΔR vs. field of these devices with a perpendicular applied field is shown in Figure 8 where a small but distinct GMR of 0.03% is observed at room temperature.

The shape of the *MR* curve matches that of the hysteresis loop for the sample, where domain rotation occurs to the antiparallel state, followed by a rapid nucleation reversal. This explains the asymmetry of the GMR peak, with a slow approach to a high resistance state, but a rapid return to the low resistance state at a definite field.

## 4. Materials and Methods

Epitaxial Heusler-alloy films have been deposited using ultrahigh vacuum (UHV) sputtering or molecular beam epitaxy (MBE) with precise control of compositions to satisfy their stoichiometry. For the UHV sputtering, compositions of targets need to be carefully optimised or combinatorial sputtering needs to be employed. For UHV MBE, simultaneous deposition is typically used on a single-crystal substrate. Polycrystalline Heusler-alloy films, on the other hand, have been grown by high-target utilisation sputtering system (HiTUS) [116]. In both of the films, substrate heating is often utilised to assist crystalline formation of the Heusler alloys. Here, the sputtering has higher energy on the materials to be deposited than those for UHV MBE by almost three orders of magnitude, allowing for the deposited films to be atomically well-mixed to form complex crystalline structures, as described in Section 2.1.1.

The deposited films have been characterised structurally and magnetically. The crystalline structures of the films are determined by X-ray diffraction (XRD, Rigaku, Tokyo, Japan) with chemical composition analysis, such as energy dispersive X-ray spectroscopy (EDX) and electron energy loss spectroscopy (EELS). Cross-sectional transmission electron microscopy (TEM, JEOL, Tokyo, Japan) is also used to investigate atomic ordering and interfacial structures of the films. The magnetisation loops of the films are measured using a vibrating sample magnetometer (VSM, MicroSense, Lowell, MA, USA) or similar methods under elevating temperatures. Temperature-dependent electrical resistivity measurements can also reveal the detailed scattering mechanism by defects in the films [52]. The half-metallicity can be determined by point-contact Andreev reflection (PCAR) [32] and infrared photoexcitation [42]. Additionally, X-ray magnetic circular dichroism (XMCD) with synchrotron radiation can reveal spin and orbital moments per constituent atoms [34].

The optimised Heusler-alloy films can be used as a ferromagnetic electrode in TMR and GMR junctions. The TMR junctions can be characterised using current-in-plane tunneling (CIPT) [117], which provides accurate TMR ratios. The GMR junctions can also be analysed by a conventional four-terminal method in a current-in-the-plane (CIP) configuration, which is more than one order of magnitude smaller than that in a CPP configuration. Therefore, these films are required to be patterned into nanometre-scale pillar junctions by electron beam lithography (EBL) and Ar-ion milling. The TMR or GMR junctions are patterned into nanopillars by EBL and Ar-ion milling, followed by the insulator deposition to isolate the pillars. For preparing the sample for electrical measurement, the top and the bottom of the pillar were connected to large contact pads via two-step lithography. Finally, smaller contacts were fabricated by EBL, and then the large contact pads were made by optical lithography.

## 5. Conclusions

The importance of the development of half-metallic ferromagnetic films for room-temperature operation has been increasing significantly. Among candidates for them, Heusler-alloy films have the greatest potential and have attracted intensive attention. Even though the bulk of the Heusler alloys have already been proven to be half-metallic, the film form still suffers from the interfacial atomic disorder against the neighbouring tunnelling barrier or non-magnetic spacer in magnetic tunnel or giant magnetoresistive junctions, respectively. For further improvement, the optimisation of growth conditions and the selection of better seed or barrier/spacer layers are crucial. Such improvement can also induce perpendicular magnetic anisotropy for the device miniaturisation. MgO- or bcc-seed-induced perpendicular anisotropy may lead to the Heusler-alloy films to satisfy the requirements for the next-generation spintronic devices.

## Figures and Tables

**Table 1:** List of measured saturation magnetisation (*M*_S_) and perpendicular magnetic anisotropy (PMA) for major Heusler alloys.

Heusler Alloy	*M*_S_ (mu/cm^3^)	PMA (erg/cm^3^)
MnGa [113]	200	3 × 10^6^
Co_2_FeAl/MgO [114]	731	1.9 × 10^6^
V/Co_2_FeSi	700	1.75 × 10^3^
W/WO_x_/Co_2_FeSi	400	4.00 × 10^3^
W/Co_2_FeSi	600	–
